# First successful use of ampicillin-sulbactam for rare *Streptococcus agalactiae*-associated peritonitis in peritoneal dialysis: a case report and literature review

**DOI:** 10.3389/fmed.2025.1652738

**Published:** 2025-09-02

**Authors:** Carlos Berrocal, J. M. Quintero-Romero, Luz Mejia, A. Zapata-Aristizábal, H. A. Nati-Castillo, Alice Gaibor-Pazmiño, Juan S. Izquierdo-Condoy

**Affiliations:** ^1^Departamento de Medicina Interna, Universidad del Valle, Cali, Colombia; ^2^Facultad de Medicina, Corporación Universitaria Empresarial Alexander von Humboldt, Armenia, Colombia; ^3^Interinstitutional Internal Medicine Group (GIMI 1), Departamento de Medicina Interna, Universidad Libre, Cali, Colombia; ^4^One Health Research Group, Universidad de las Americas, Quito, Ecuador

**Keywords:** chronic kidney disease, peritoneal dialysis, *Streptococcus agalactiae*, ampicillin-sulbactam, refractory peritonitis

## Abstract

**Introduction:**

Peritonitis is a major complication of peritoneal dialysis, most often caused by gram-positive cocci. *Streptococcus agalactiae* (Group B Streptococcus) is an exceptionally rare pathogen in this context.

**Case presentation:**

We describe a 64-year-old man with end-stage renal disease on long-term PD who developed refractory peritonitis due to *S. agalactiae*. Peritoneal fluid analysis revealed 525 leukocytes/μL (74% polymorphonuclear cells) and Gram-positive cocci. Cultures confirmed *S. agalactiae*, fully susceptible to all tested antibiotics. Despite intraperitoneal vancomycin, the patient showed no clinical improvement. Because of limited intraperitoneal antibiotic availability, intravenous ampicillin–sulbactam (1.5 g every 12 h) was initiated, combined with prophylactic oral fluconazole. Clinical resolution was achieved after 10 days, followed by four days of oral therapy. The peritoneal catheter was subsequently removed, and the patient transitioned to intermittent hemodialysis.

**Conclusion:**

This case represents the first documented success of intravenous ampicillin–sulbactam for *S. agalactiae*–associated peritonitis in PD. It expands the therapeutic options for this rare and challenging infection and highlights the importance of culture-guided management and adaptive treatment strategies, particularly in resource-limited settings where conventional intraperitoneal therapies may be unavailable.

## 1 Introduction

Peritonitis remains a frequent and serious complication among patients undergoing peritoneal dialysis (PD), contributing significantly to morbidity, catheter loss, ultrafiltration failure, transition to hemodialysis, and mortality, with reported death rates ranging from 2 to 6% (1, 2). Despite this clinical burden, peritonitis is largely preventable through strict hygienic practices, proper system handling, and adherence to follow-up protocols (2, 3).

From a microbiological perspective, the etiology of PD-associated peritonitis is well characterized. The majority of cases are caused by gram-positive cocci (80–95%), particularly skin-colonizing organisms such as *Staphylococcus* spp., *Streptococcus* spp., and *Enterococcus* spp. (3, 4). These pathogens typically gain access to the peritoneal cavity through touch contamination or via the catheter exit site. Gram-negative bacilli—including *Escherichia coli*, *Klebsiella* spp., and *Pseudomonas aeruginosa*—account for fewer than 30% of cases, yet they are often associated with more severe clinical presentations and higher hospitalization rates. Anaerobic organisms are responsible for fewer than 3% of cases, usually in the context of intra-abdominal pathology or bowel perforation (3, 5).

Within the group of gram-positive cocci, *Streptococcus* species represent a notable subgroup, accounting for approximately 11.7% of PD-associated peritonitis cases. The most frequently isolated are alpha-hemolytic species, predominantly viridans group streptococci such as *S. sanguis*, *S. mitis*, *S. salivarius*, *S. bovis*, and *S. constellatu* (6). In contrast, beta-hemolytic species such as *Streptococcus agalactiae* (Group B Streptococcus) are exceedingly rare in PD patients, with only isolated case reports available. A comprehensive literature review across multiple databases identified nine documented cases worldwide (7–15).

This infection is clinically important because commonly used empirical antibiotics may be less effective against *S. agalactiae*, partly due to its ability to form biofilms on PD catheters, which can impair antibiotic penetration and limit therapeutic concentrations.

Here, we report a case of PD-associated peritonitis caused by *S. agalactiae*, emphasizing its clinical presentation and course.

## 2 Case presentation

A A 64-year-old male with end-stage chronic kidney disease (CKD) secondary to hypertensive nephrosclerosis, maintained on peritoneal dialysis for the past 10 years, presented with a two-week history of abdominal pain, nausea, occasional vomiting, and infrequent loose stools. On Day 5 of illness, he sought care at the renal unit, where a peritoneal fluid evaluation was performed.

Macroscopic examination of the effluent revealed purulent characteristics, with marked changes in color and appearance. Microscopic analysis showed 525 white blood cells/μL, predominantly polymorphonuclear cells (74%), and Gram staining demonstrated Gram-positive cocci (Table 1). Based on these findings, empirical intraperitoneal antibiotic therapy with vancomycin (30 mg/kg every 24 h) was initiated and continued from Day 5 to Day 12.

**Table 1:** Cytochemical analysis of peritoneal fluid.

Parameter	Result
Color	Yellow
Appearance	Slightly turbid
Red blood cell count	Not observed
White blood cell count	525 cells/μL
Neutrophils	74% (394 cells/μL)
Lymphocytes	25% (131 cells/μL)

Peritoneal fluid cultures, incubated on aerobic blood agar at 35–37°C, yielded *Streptococcus agalactiae* after 24 h. The isolate was fully susceptible to all antibiotics tested, while two sets of blood cultures remained negative. Despite targeted intraperitoneal vancomycin, the patient showed no clinical improvement after seven days, with persistent abdominal pain and cloudy peritoneal fluid.

He was subsequently referred to a tertiary care center for advanced management. On admission, vital signs were stable. Given the limited availability of intraperitoneal antibiotics—particularly narrow-spectrum agents—intravenous ampicillin–sulbactam (1.5 g every 12 h) was initiated on Day 15, together with oral fluconazole (200 mg every 8 h) as prophylaxis against fungal peritonitis. A double-lumen jugular catheter was placed, and the peritoneal dialysis catheter was removed on Day 21, following delayed authorization from the national health insurance system.

**Figure 1:**
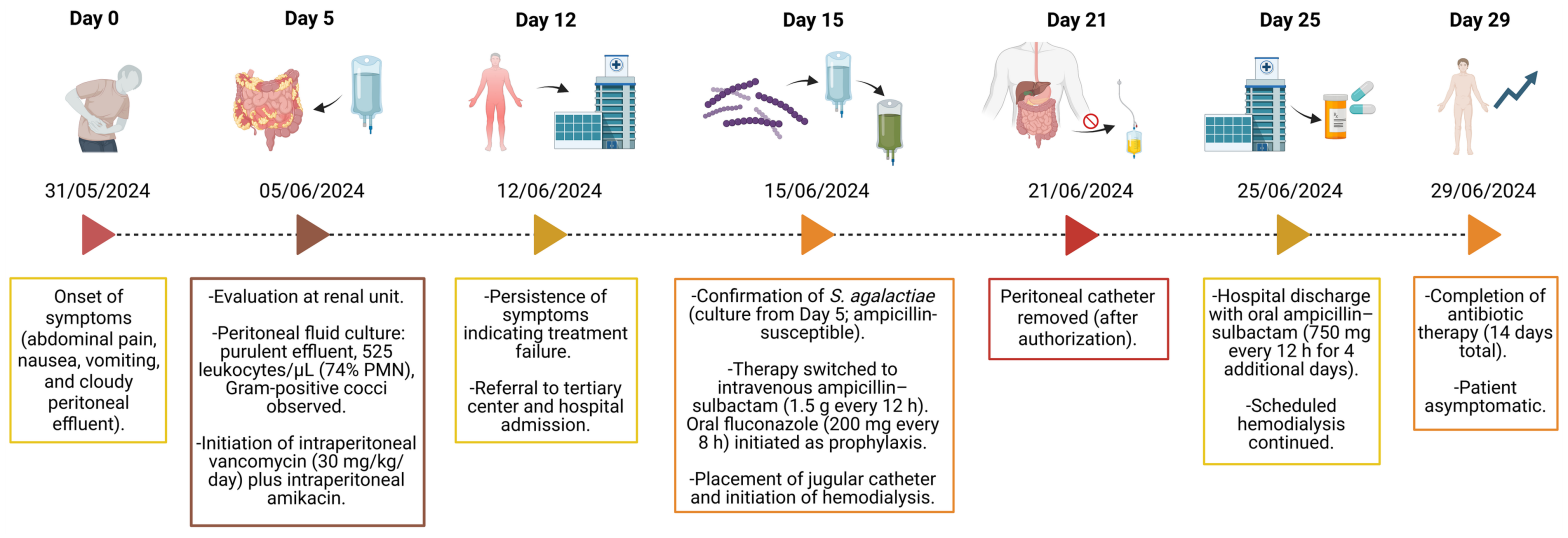
Timeline of patient evolution.

After 10 days of intravenous therapy, the patient demonstrated clinical improvement and was discharged on oral ampicillin–sulbactam (750 mg every 12 h) for an additional four days. At discharge, he was asymptomatic, with complete resolution of abdominal pain and normalization of peritoneal effluent appearance. The patient transitioned to intermittent hemodialysis and remained under follow-up care (Figure 1).

## 3 Discussion

We report a case of a male patient with end-stage chronic kidney disease who developed peritonitis caused by *S. agalactiae*. Peritonitis remains a common yet preventable complication of PD (1). Diagnosis relies on clinical presentation and peritoneal fluid analysis, with leukocyte counts exceeding 100 cells/μL (after two hours) and more than 50% polymorphonuclear cells (4). In our patient, the leukocyte count was 525 cells/μL with 74% polymorphonuclear cells, meeting diagnostic criteria. Nevertheless, up to 10% of cases may present with lower leukocyte counts, underscoring the importance of clinical judgment (15). Blood cultures are recommended when sepsis is suspected and are positive in up to 11% of cases (16).

In this case, *S. agalactiae*—a gram-positive, catalase-negative, beta-hemolytic facultative anaerobe—was isolated. This organism is classically associated with neonatal meningitis and sepsis, puerperal sepsis in pregnant women, and diverse adult infections including pneumonia, osteomyelitis, and urinary tract infections. The risk of severe infection increases with comorbidities such as advanced age, diabetes mellitus, obesity, chronic kidney disease, and immunosuppression (7, 17). Notably, our patient had chronic kidney disease as the sole identifiable risk factor. While *S. agalactiae* is recognized as a cause of multiple infections in the general population, reports in PD patients remain confined to peritonitis.

*S. agalactiae*–associated peritonitis in PD is exceedingly rare, with only nine cases reported worldwide to date (7–14) (Table 2). This case represents the second report from Latin America and the first among Hispanic American countries. Among published cases, 78% (7/9) occurred in adults, with equal sex distribution, while two cases (22%) were pediatric (9). Septic shock was documented in 33% (3/9)—cases (8), (9), and (11)—including one fatal outcome despite broad-spectrum therapy, for an overall mortality of 11% (1/9) (11). Bacteremia was confirmed in two patients (8, 14). Catheter removal or replacement was required in 22% (2/9)—replacement in case (9) and removal after three weeks in case (7)—although most reports did not specify catheter management, limiting conclusions regarding catheter preservation. Symptom resolution, when documented, generally occurred within 14 days of initiating antibiotics in five cases (7, 8, 12–14), with rapid improvement (~72 h) in two (13, 14). The longest hospitalization (40 days, case 12) was attributable to antibiotic-induced bone marrow aplasia rather than persistent infection. Clinical and microbiological cure with initial therapy—without the need for further intervention—was reported in 67% (6/9) (7, 10, 12–15). Non-responders included those requiring catheter replacement due to poor response (9), catheter removal after prolonged therapy (7), or death despite broad-spectrum treatment (11). In two reports, the source was traced to vaginal carriage of *S. agalactiae* (7, 14), supporting the hypothesis that colonization of the genital and anal tracts contributes to pathogenesis. Furthermore, colonization studies suggest possible person-to-person transmission, with higher carriage rates observed in older adults (18, 19).

**Table 2:** Reported cases of *Streptococcus agalactiae* peritonitis associated with peritoneal dialysis.

Author and year	Country	Age	Sex	CKD etiology	PD duration	Clinical presentation	Cytochemical characteristics (Cell count)	Antimicrobial therapy	Susceptibility	Outcome
Schroder et al., 1991 (9)	Netherlands	13 months	Male	Renal dysplasia and ureteral valves	2 months	8 h of anorexia, fever, and cloudy dialysis effluent	No cell count data; Gram stain: gram-positive cocci	Tobramycin IP and cefalotin IP	Sensitive to cefalotin	Dialysis catheter removed due to poor antibiotic response. Resuscitated from cardiac arrest; complications included tetraplegia and severe mental retardation.
		5 years	Male	Unknown	3 years	One day of fever, abdominal pain, and cloudy dialysis effluent	No cell count data; Gram stain: gram-positive cocci	Tobramycin IP and cefalotin IP	Sensitive to cefalotin	Recovered within 48 h of treatment
Borra et al., 1992 (11)	USA	52 years	Male	Unspecified glomerulonephritis	25 days	Fever, mild abdominal pain, and cloudy dialysis effluent	>100 cells/mm^3^ (majority polymorphonuclear)	Peritoneal lavage, vancomycin IV, and IM gentamicin; later switched to vancomycin IV, gentamicin IV, and clindamycin IV	Postmortem analysis: group B *Streptococcus* sensitive to vancomycin and gentamicin	Patient died
Yinnon et al., 1993 (8)	USA	63 years	Male	Membranous glomerulopathy	4 months	One day of lower abdominal pain and cloudy dialysis effluent	52,500 cells/mm^3^ of nucleated cells, predominantly neutrophils	Vancomycin (30 mg/kg loading dose) IV and gentamicin IP for one week	Sensitive to penicillin, clindamycin and vancomycin	Survived; peritoneal dialysis was not interrupted
Scanziani et al., 1999 (10)	Italy	23	Female	Renal dysplasia, chronic pyelonephritis	6 months	12 h of Abdominal pain, vomiting, chills; and cloudy dialysis affluent.	760 cells /mm^3^ and 85% segmented neutrophilis. Gram stain: gram positive cocci.	Cephalotin IP and netilmicin IP	Sensitive to ampicillin, cephalotin, penicillin and Vancomycin	Recovery at 3 days.
Pagniez et al., 1995 (14)	France	25	Male	Membranous nephropathy	7 years	Abdominal pain, stupor; temp 39.5°C and cloudy dialysis affluent.	The first bag was clear, with 2 cells /mm^3^; the second was cloudy with 1,400 cells/mm^3^	Piperacillin IP, cephalothin IP + IV loading Piperacillin and cephalotin. Cephalothin was stopped when culture results were known.	Sensitive to penicillin	Recovery at 3 days. Discharged 7 days later on oral rifampin.
Liakopoulos et al., 2004 (7)	Germany	27 years	Male	Membranoproliferative glomerulonephritis type 1	17 months	Severe abdominal pain, chills, and fever	Data not reported	Ceftazidime, IP tobramycin IP, and vancomycin IV; 14 days of treatment	Data not reported	Abdominal pain improved within 12 days; white cell counts in effluent normalized by day 14
De los Santos et al., 2010 (12)	Brazil	52 years	Female	Alport syndrome with deafness and CKD	1 year, 6 months	Diffuse abdominal pain	1,500 cells/mm^3^; 92% neutrophils	Gentamicin IP and cefalotin IP; later switched ceftazidime IP, tobramycin IP and IV vancomycin for 3 weeks; catheter removed; switched to hemodialysis	Sensitivity not reported	Discharged 40 days after admission due to hematologic complications (bone marrow aplasia)
Güngör et al., 2012 (13)	Turkey	39 years	Female	Vesicoureteral reflux	4 years	Abdominal pain, nausea, vomiting, and cloudy dialysis effluent	>1,000 cells/mm^3^	Ceftazidime IP and cefazolin IP; vancomycin IP added the following day; then ceftazidime discontinued, cefazolin continued; treatment lasted 4 days	Sensitive to penicillin	Symptoms resolved within 14 days

GBS, Group B streptococcus; RPGN, rapidly progressive glomerulonephritis; IP, intraperitoneal; IV, intravenous; CKD, chronic kidney disease.

Empiric therapy in reported cases most often included a third-generation cephalosporin (78%), frequently combined with an aminoglycoside (56%) and, in severe presentations, vancomycin (44%). Intraperitoneal administration was the preferred route, occasionally preceded or supplemented by an intravenous loading dose or short IV course (8, 9, 11, 13, 14). Once culture results were available, therapy was typically de-escalated to narrow-spectrum beta-lactams: first-generation cephalosporins (44%), ampicillin (22%), or penicillin G (11%). A third-generation cephalosporin was continued in 22%, while piperacillin was used in one case. Treatment duration ranged from 7 to 40 days, with most favorable outcomes achieved after 10–14 days of intraperitoneal therapy. As little as 7 days of treatment sufficed in one case (14) with rapid resolution, whereas prolonged therapy was required in patients with catheter-related complications or antibiotic-related adverse events.

However, in this case, the patient failed to respond to intraperitoneal vancomycin. At the time, intraperitoneal antibiotics—particularly narrow-spectrum agents—were not available in our formulary. This limitation reflects the realities of many low- and middle-income healthcare systems, where therapeutic options are constrained by access, formulary restrictions, and administrative processes. Such circumstances often compel clinicians to adapt treatment pathways. We initiated intravenous ampicillin–sulbactam, achieving therapeutic success. This is the first reported case of *S. agalactiae* peritonitis in PD successfully treated with this regimen. Ampicillin–sulbactam reaches therapeutic concentrations in peritoneal fluid (20). Following intravenous administration (0.5 g/0.5 g), peritoneal fluid concentrations of ampicillin and sulbactam reach approximately 7 μg/mL and 14 μg/mL, respectively. Because peritoneal dialysis clears these drugs less efficiently than hemodialysis, dose adjustment is required to avoid accumulation and toxicity (21). In this case, we administered intravenous ampicillin–sulbactam for 10 days, selected based on clinical improvement, delayed catheter removal, and resolution of peritoneal symptoms, followed by a four-day step-down course of oral amoxicillin–clavulanate. In the absence of clear evidence regarding optimal treatment duration, a prolonged course was chosen to ensure sustained bactericidal concentrations in the peritoneal fluid.

Prolonged antibiotic therapy increases the risk of fungal infection, necessitating antifungal prophylaxis (2, 3). Additionally, refractory peritonitis requires peritoneal catheter removal to minimize complications. In our case, catheter removal was delayed due to the need for prior authorization from the national health insurance system, a common administrative barrier in our setting.

This case underscores the importance of individualized therapeutic approaches in rare infections such as *S. agalactiae* peritonitis. The successful use of intravenous ampicillin–sulbactam under resource constraints broadens the therapeutic landscape for patients unresponsive to standard therapy and highlights a practical option for centers in resource-limited environments. Further case reporting and studies are warranted to refine management strategies for this uncommon but clinically significant infection.

## 4 Conclusion

This case illustrates the clinical and therapeutic challenges of managing rare infections such as *Streptococcus agalactiae*–associated peritonitis in peritoneal dialysis patients. It emphasizes the importance of rapid microbiological diagnosis to guide targeted therapy and the need to adapt treatment strategies when standard approaches fail. Given the variable susceptibility profiles and the risk of therapeutic failure, culture-guided management remains essential to ensure timely de-escalation or modification of antimicrobial regimens. The successful use of intravenous ampicillin–sulbactam in this refractory case broadens the therapeutic options for *S. agalactiae* peritonitis and provides a practical alternative when conventional empirical agents, such as vancomycin, prove ineffective—particularly in resource-limited settings.

## Data Availability

The original contributions presented in the study are included in the article/supplementary material, further inquiries can be directed to the corresponding author/s.
